# Cage enrichment to minimize aggression in part-time group-housed female breeding rabbits

**DOI:** 10.3389/fvets.2024.1401021

**Published:** 2024-06-04

**Authors:** Liesbeth G. W. Van Damme, Nusret Ipek, Jan Verwaeren, Evelyne Delezie, Frank A. M. Tuyttens

**Affiliations:** ^1^Animal Sciences Unit, ILVO, Melle, Belgium; ^2^Department Data Analysis and Mathematical Modelling, Ghent University, Ghent, Belgium; ^3^Department of Veterinary and Biosciences, Faculty of Veterinary Medicine, Ghent University, Merelbeke, Belgium

**Keywords:** does, kits, skin injuries, agonistic behavior, activity

## Abstract

In most rabbit farms, breeding does kindle and nurse their kits in single-litter cages throughout their entire reproduction cycle. However, the protective behavior can lead to aggressive displays and injuries when the does are housed in groups. This study aimed to evaluate cage enrichment for reducing the agonistic behavior in part-time group-housed does. A total of eighty does with their 22-day-old kits were allocated to 20 multi-litter cages, with each cage housing four does and their litters for 10 days. Each multi-litter group was subjected to one of four treatments: alfalfa blocks as distraction material (A), wooden panels underneath the platforms (P), both alfalfa and wooden panels (AP), or no extra enrichment (controls, C). This experiment was replicated for three consecutive reproduction cycles. The skin injuries of the does and the kits were scored with a tagged visual analog scale before grouping and at one, three, six, eight, and 10 days after grouping. Computer vision techniques were used to continuously monitor rabbit activity and agonistic behavior (aggression and fleeing/chasing) during the first 24 h after grouping, specifically during light hours. During the first day in the group, 67.2% of the does and 13.4% of the kits acquired new injuries. This increased to 82.0 and 33.2%, respectively after 10 days in the group relative to the onset of grouping. The injury scores of the does increased toward the sixth day after grouping compared to the first (*p* < 0.001) and were highest on the tenth day for the kits (*p* < 0.001). On all the observation days, the number of injured does was higher in C compared to A (*p* = 0.04) and AP treatment (*p* = 0.005). There were no other treatment effects observed on the doe or kit skin injuries. Rabbit activity was highest after grouping but decreased after the first and second days (*p* < 0.001). The agonistic interactions between the does involved more fleeing/chasing behavior (62.0%) rather than aggression (38.0%). Although hierarchy fights are likely when unacquainted does are group-housed, the many animals that sustained injuries and the high injury scores confirm that part-time group housing for does is challenging and possibly inevitable. This study has shown that alfalfa, with or without wooden panels, can slightly reduce the number of injured does.

## 1 Introduction

Society increasingly expects social farm animals, including rabbits (*Oryctolagus cuniculus*), to be housed in groups, preferably in housing systems already adapted to the animals’ species-specific needs. In Belgium and the Netherlands, weaned meat rabbits are housed in groups in enriched multi-litter cages. In contrast, breeding does kindle and nurse their kits in single-litter cages throughout their entire reproduction cycle. Breeding does may also benefit from additional space and social contact with other does (1), therefore, housing small groups of does with their litters in multi-litter cages has been proposed and investigated (2). Unfortunately, aggressive behavior among does results in injuries, stress, and reduced reproductive performance (3–5). As part of the aggression seems related to the maternal protective behavior during early lactation (6), part-time group housing has been proposed as an alternative (7, 8). In this system, does are housed in single-litter cages during the first weeks after kindling and housed in groups when maternal protective behavior is reduced and the kits are older and more independent from the mother. Although reproductive performances improved compared with continuous group housing (8, 9), the doe-doe aggression, mainly due to hierarchy fights, remained an important and yet-to-overcome problem (5, 10).

The enrichment of cages to minimize aggressive behavior and its negative impacts has been suggested as a potential solution. The provision of suitable enrichment for hiding and fleeing not only raises a sense of security (11) but also offers an escape route from aggressive pursuers. In a review of group housing for sows, Schubbert et al. (12), proposed the use of visual barriers to prevent aggressive behavior. The barriers reduced the number of aggressive interactions by approximately one-third within the first 12 h after grouping. Verdon et al. (13) and Luescher et al. (14) also reported on the effectiveness of visual barriers in group-housed sows for decreasing aggressive interactions and skin injuries, while providing visual isolation from group mates. The ability to hide was sufficient to stop a fight among weaned pigs (*Sus scrofa domesticus*) (15). While observing the group-housed breeding does, Rommers et al. (16) noted the elevated platforms and pipes reduced the severity of doe injuries after grouping.

Other types of enrichment, such as those that focus on foraging needs, may also affect the level of aggression, albeit in a less direct way. In group-housed sows and weaned pigs, distraction-based enrichment (mushroom compost or mineral blocks) showed an effective reduction in aggressive displays (17, 18). Although the information on feed-related enrichment for on-farm breeding is limited, such items may potentially reduce the doe-doe agonistic behavior by providing distraction at the onset of grouping. Furthermore, as wild rabbits can spend the majority of their time searching for and eating their food (19, 20), feed enrichment also aligns with their natural behavior.

The present study aims to evaluate the effects of the two types of cage enrichment and their combined effect on the agonistic behavior of does and to evaluate skin injuries in breeding the does housed in groups with their kits from 22 until 32 days post-partum (pp). The first type of cage enrichment aimed to reduce the frequency of agonistic behavior by providing hiding panels so rabbits could avoid aggressive group mates. In the second type of enrichment, the blocks of pressed alfalfa were added to the cages at the start of group housing as a novel distracting feed source. The alfalfa blocks were assumed to keep the rabbits occupied instead of engaging in agonistic behavior. The effect of both types of enrichment on doe reproductive performance has been reported in the study by Van Damme et al. (21) (under review).

## 2 Materials and methods

The protocols and procedures for this trial were approved by the Ethics Committee for the Use of Animals in Research (EC 2021/389) of Flanders Research Institute for Agriculture, Fisheries and Food (ILVO).

### 2.1 Animals and experimental design

The trial was conducted on a commercial rabbit farm in Flanders (Belgium) from June to October 2021. A total of 80 random Hyla does (Sevremoine, France, and Valli del Pasubio, Italy), in their 3rd to 5th parity, were selected for the experiment, from among the female breeding rabbits present in the farm. In addition, 25 does were selected as spare does to replace deceased or non-pregnant does during the experiment. According to the farm’s practice, does were housed in single-litter cages (100 × 50 cm) with elevated platforms (50 × 30 cm), an open roof, a plastic slatted floor, and a wooden gnawing block. All does were artificially inseminated 10 days after parturition. This resulted in a 42-day reproduction cycle, which included a gestation duration of 32 days. Three days prior to the expected birth of litters, the does were provided with a nest box and nesting material (flax and wood shavings). According to common farm practice, kits were cross-fostered to create homogenous litters 1 day postpartum (pp). Each doe was assigned 11 kits for nursing. The surplus of kits was euthanized (*N* = 227), according to the standard practice of the farm.

**Figure 1:**
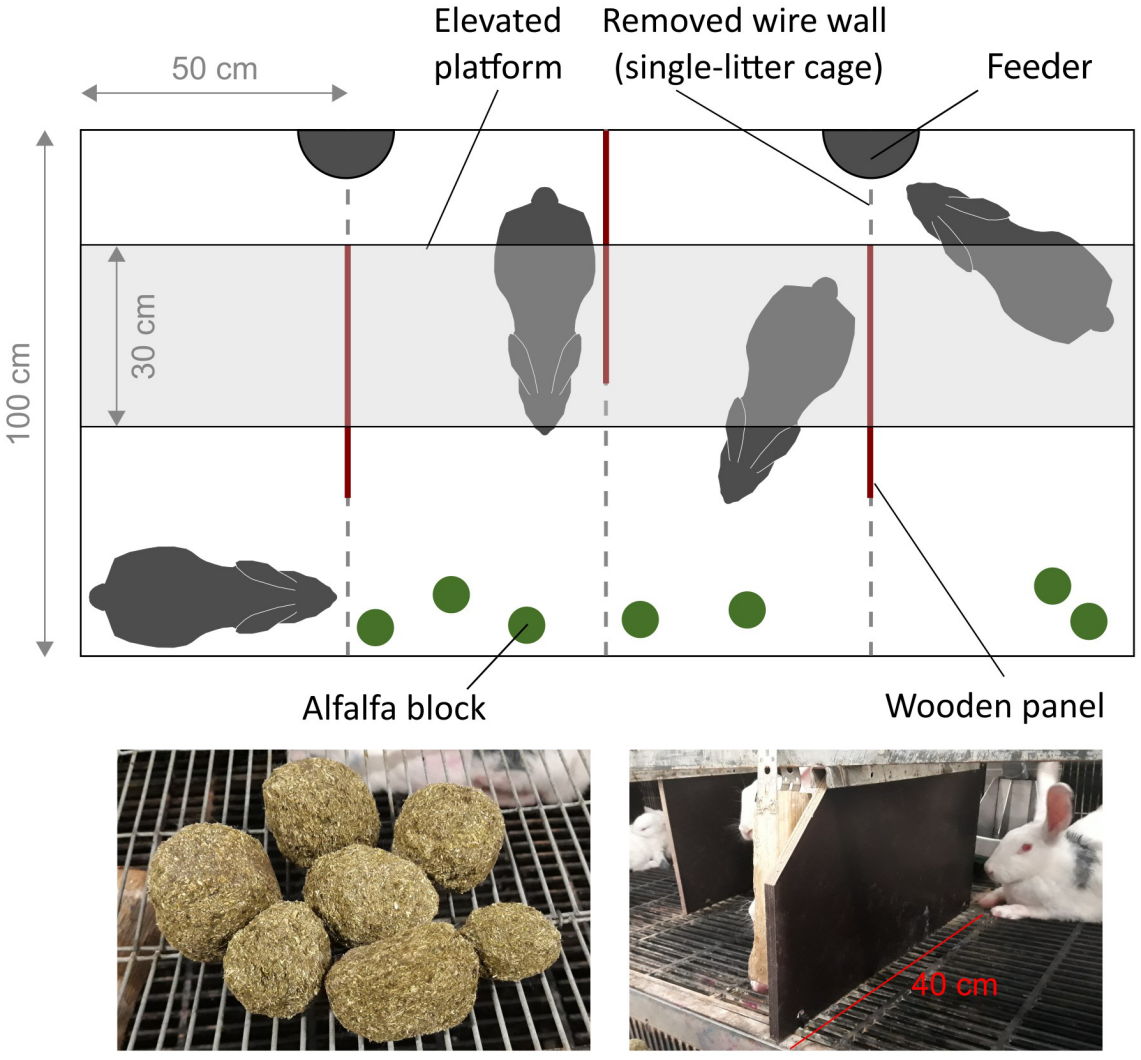
Multi-litter housing of four does and their kits (not shown) between day 22 and 32 pp. Three wooden panels were provided underneath the elevated platform in the P and AP treatment. The two outer panels were aligned with the backside of the platform, and the middle panel was pressed against the back of the cage, separating the two feeding areas on the left and right of the middle panel. Free passage was possible on both sides of the panels except the middle panel (which could only be passed at the front or by jumping over the panel). Sharp corners of the outer panels were chamfered for safety. Small blocks of pressed alfalfa were provided in the front of the cage in the alfalfa and alfalfa plus panels treatments. Water nipples were located at the back of the cage and gnawing blocks were attached to the front side of the platform (not shown in the figure).

For the experiment, all 80 does were housed in 20 multi-litter cages (four does per cage) with their kits between day 22 and 32 pp. Multi-litter cages were created by removing wire walls between four adjacent single-litter cages. The experiment was repeated for three consecutive reproduction cycles. Each newly created group was assigned one of the following treatments in a completely randomized block design (*N* = 15 multi-litter cages per treatment): provision of small pressed alfalfa blocks as distraction enrichment (A), three wooden panels attached underneath the platforms, visually separating the cage into four areas (P), both alfalfa and wooden panels (AP), or no extra enrichment (controls, C; Figure 1). The experimental group-housing phase lasted for 10 days, ending at 32 days pp. when the does and their kits were moved to a different compartment. During the duration of the three experimental reproduction cycles, the does were never assigned to the same multi-litter cage, group mates, and treatments more than once. All animals were in good body condition and showed no signs of sickness before the experiment. During the experiment, deceased, non-pregnant, sick, or severely injured does were replaced with does from the spare group when housed in the single-litter cages. The alfalfa blocks were replenished in all multi-litter cages during the experiment and were available *ad libitum*.

At the end of each experimental group-housing phase (32 days pp), does and their kits were housed in multi-litter cages in a separate compartment (same groups as experimental phase). This was not a part of the experiment but in line with the farm’s management. Three days later, and according to the farm’s management, the does were moved back to the single-litter cages (weaning, day 35 pp) to prepare for their next litter. The kits remained in the multi-litter cages until slaughter age (10–11 weeks).

During the experiment, all animals on the farm were provided with commercial rabbit feed (Quartes nv, Deinze, Belgium), free access to fresh water (nipple drinkers), and a wooden gnawing block. The illumination cycle was set at 12 L:12D, except for 7 days before artificial insemination when it was changed to 16 L:8D. Light intensity was higher than 40 lux during the light hours. The temperature was set at 20–21°C and relative air humidity ranged between 60 and 75%.

### 2.2 Data collection

#### 2.2.1 Skin injuries

**Figure 2:**
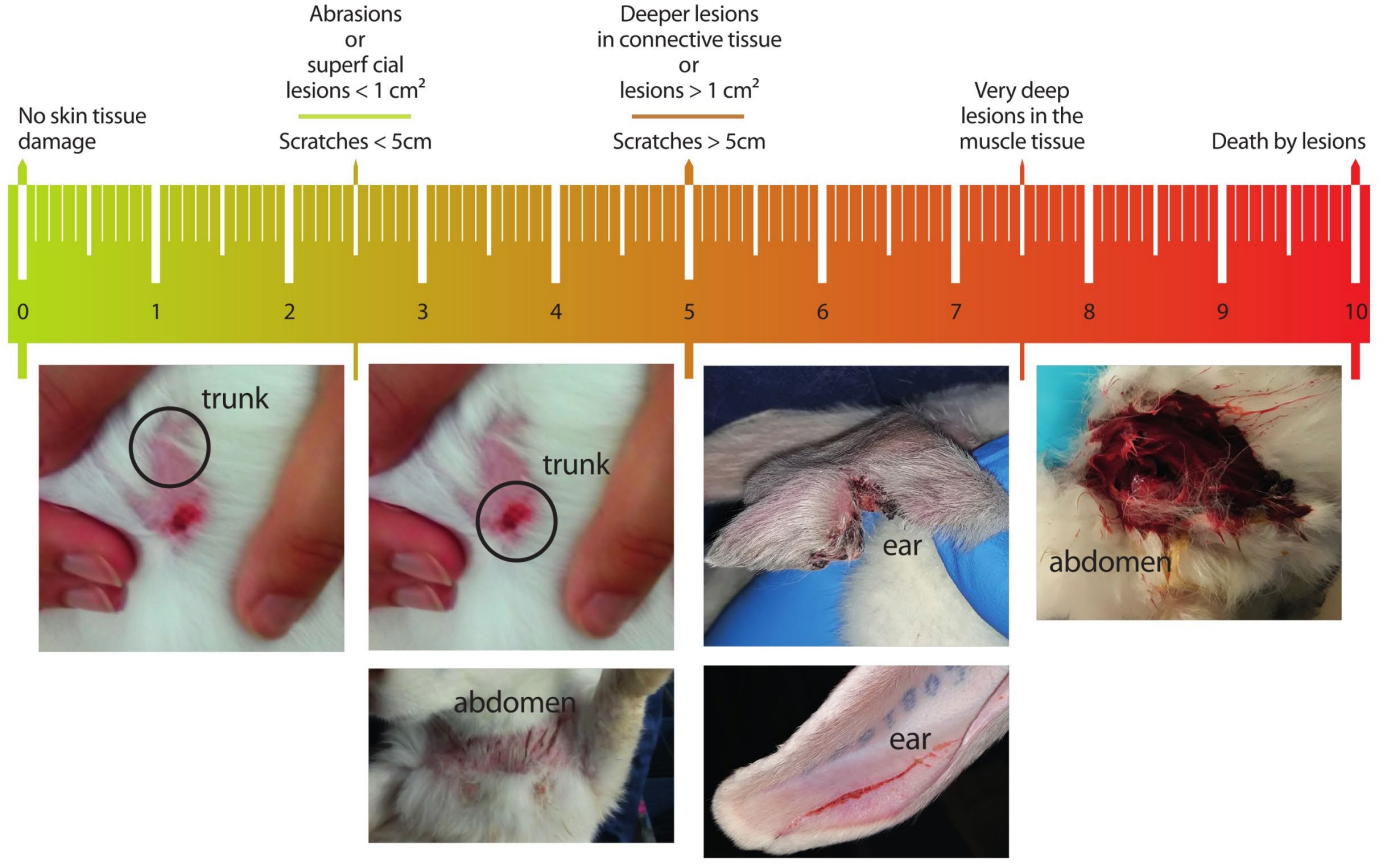
Tagged visual analog scale for doe and kit skin injuries ranging from 0 to 10 [adapted from Van Damme et al. (21)]. Pictures below the scale provide a visual presentation of the description corresponding to the indicated range (e.g., the leftmost picture belongs to the range 0–2.5, while the rightmost picture to the range 7.5–10). Upper tags [adapted from Andrist et al. (2)] indicate descriptions to guide the observer along the scale.

Prior to the group housing on day 22 pp., both does and kits were checked for skin injuries. Each observed skin injury was scored with a tagged visual analog scale (t-VAS) ranging from 0 to 10 [Figure 2, adapted from Van Damme et al. (21)]. A higher score on the scale corresponded with a higher severity of the injury. For each injury observed, the severity score and the location on the body (ears, eyes, nose, head, trunk, front paws, hind paws, tail, abdomen, or genitals) were noted. Scores of injuries on sensitive body areas (eyes, nose, and genitals) were multiplied by two. After grouping, the rabbits were checked again for skin injuries on days 1, 3, 6, 8, and 10. No distinction was made between fresh and old wounds, but the injuries that were present before group housing began were ignored (because we were interested in newly acquired injuries since the start of the group-housing phase). After data collection, severity scores of individual injuries were summed for each doe and kit on each observation day. Before the experiment, four observers were trained to use the t-VAS followed by an inter-rater reliability test. Each observer scored 64 pictures of skin injuries of rabbits, ranging from mild to severe, resulting in an intraclass correlation coefficient (calculated in R 4.1.2 with the ICC package) of 0.75 (95% confidence interval 0.67–0.83). This is considered good reliability between observers (22). During the experiment, observers were completely aware of the treatments. Although the protocol provided a strict step-by-step guideline for scoring the injuries, the observer bias could not be fully excluded.

#### 2.2.2 Behavior and activity

Infrared cameras were installed above all the multi-litter cages. The does were sprayed on the back with unique marks with black paint for individual recognition. The animals were video-recorded continuously from the start until the end of group housing (day 32 pp). The optical flow estimation was used to clip the video footage into smaller fragments during which the does move. The clips starting when at least one doe in the multi-litter cage started moving and ending when all does return to a stationary state were retained. It was during these ‘action’ clips that the does display agonistic behavior, which was the focus of this experiment. A detailed and technical description of rendering action clips and optical flow estimation is described in Ipek et al. (23). The action clips were then used for manual observation of agonistic doe behavior and automated quantification of rabbit activity (both does and kits) in the group.

The agonistic behavior between does was scored continuously by a human annotator using the action clips of the first 24 h, after grouping, for nine randomly chosen multi-litter cages per treatment. The behavior of all does was scored using Observer XT14 software (Noldus, The Netherlands). The two types of agonistic behavior were scored: aggression and flee/chase behavior (Table 1). The frequency of number of bouts was tracked for both behavioral types for each doe. A behavior was considered to have ended if more than 2 sec elapsed between two consecutive displays of the respective behavior type or if another behavior type was initiated.

**Table 1:** Ethogram used for behavioral observations of does towards other does.

Behavior group	Description
Aggression	Attack (quick movement towards another doe, neck stretched out, ears flattened, physical contact is made), threat (quick movement towards another doe, ears flattened but no physical contact is made), fight (two does get into a fight by gripping each other with their teeth and/or ripping with the hind paws), circling (two does are locked by gripping each other with the teeth, a circular movement may occur) and counter-attack (doe reacts by attacking another attacking doe)
Flee/chase	Flee (running away from approaching/attacking doe, may involve jumping over approaching/attacking doe) and chase (aggressive pursuing of another doe attempting to avoid contact)

Behavior groups were mutually exclusive. The frequency of both behavior groups was recorded for each doe.

The action clips from all multi-litter cages from the start until the end of group housing (all 10 days) were used to quantify the activity level (of both does and kits) in the groups, using the automated image-analysis tool developed and validated by Ipek et al. (23). An ‘activity score’ (continuous variable) on the group level was assigned to each action clip, with higher activity values representing more movement and thus a higher level of social unrest (0 = no activity). It must be noted that the action clips were generated based on the occurrence of agonistic behavior, meaning that activity scores indicate the extent of social unrest within the cage, rather than the rabbits’ overall activity level.

### 2.3 Statistical analysis

All analyses were conducted using the statistical software R 4.1.2 (R Foundation for Statistical Computing). In the case of deceased or removed does, data from the corresponding group was excluded for analysis, from that point onwards until the end of the remaining part of the reproduction cycle: one P treatment in cycle 1 and one A treatment in cycle 3. The data were assumed to be normally distributed based on the visual inspection of the residuals of the used models (Q-Q plots and histograms). The independent variables and their interactions were included in the models and non-significant interactions (*p* > 0.05) were excluded. In the case of a significant treatment, parity (number of successfully weaned litters), or pregnancy effect (doe pregnant at the time of measurement), a post-hoc Tukey test was performed on the estimated least squares method to evaluate all pairwise differences.

#### 2.3.1 Skin injuries

For each multi-litter cage, the percentage of does and kits with at least one skin injury was calculated for each observation day. The percentage of injured does and kits were analyzed separately with linear mixed models with treatment, time (day of observation), their interaction, pregnancy, and parity as fixed factors. The reproduction cycle and multi-litter cage were added as random factors to the models. The skin injury severity scores (summed for does and kits) were averaged for each multi-litter cage and observation day. The severity scores were log(x + 1) transformed to obtain residual normal distribution for analysis and analyzed with a linear mixed model with the same fixed and random effects.

#### 2.3.2 Behavior and activity scores

For each type of agonistic behavior between the does (aggression and flee/chase behavior) the frequency of bouts was calculated as the total number of times the behavior occurred for each doe and at each hour post-grouping (for up to 24 h). The mean values per multi-litter cage and per hour were calculated by averaging data for all does in the cage. Mean activity scores, starting from the onset of group housing at day 22 pp. until the end at day 32 pp., were averaged for each day. Prior to analysis, both behavioral and activity data were log(x + 1) transformed. The Linear mixed models were applied with treatment, time (observation hour or day), their interaction, parity, and pregnancy as fixed effects. The reproduction cycle and multi-litter cage were added as random factors.

#### 2.3.3 Correlations between skin injuries, behavior, and activity scores

Relationships between doe skin injury scores and doe agonistic behavior 24 h after grouping were tested using Spearman’s rank correlations. The same was tested between kit skin injury scores and doe behavior. Spearman’s rank correlations were also tested between doe and kit skin injury scores and activity scores on days 1, 3, 6, 8, and 10 after grouping. The mean injury and activity scores on multi-litter cage level were used for correlations.

## 3 Results

During the experiment, 27 does (33.8%) had to be replaced. Twenty does were not pregnant after insemination. Two does died before group housing of which one died due to a fatal abortion. Three does were replaced due to mastitis and two does died shortly after grouping (cause of death unknown). While housed in multi-litter cages, 1.0% of all participating kits (*N* = 23) were found deceased without a known cause of death. Another 0.6% (*N* = 14) were euthanized due to broken paws (*N* = 9), severe skin injuries (*N* = 3), or other injuries acquired shortly after grouping (*N* = 2). From among these kits, 2, 5, 5, and 2 kits were housed in C, A, P, and AP groups, respectively.

### 3.1 Skin injuries of does

**Figure 3:**
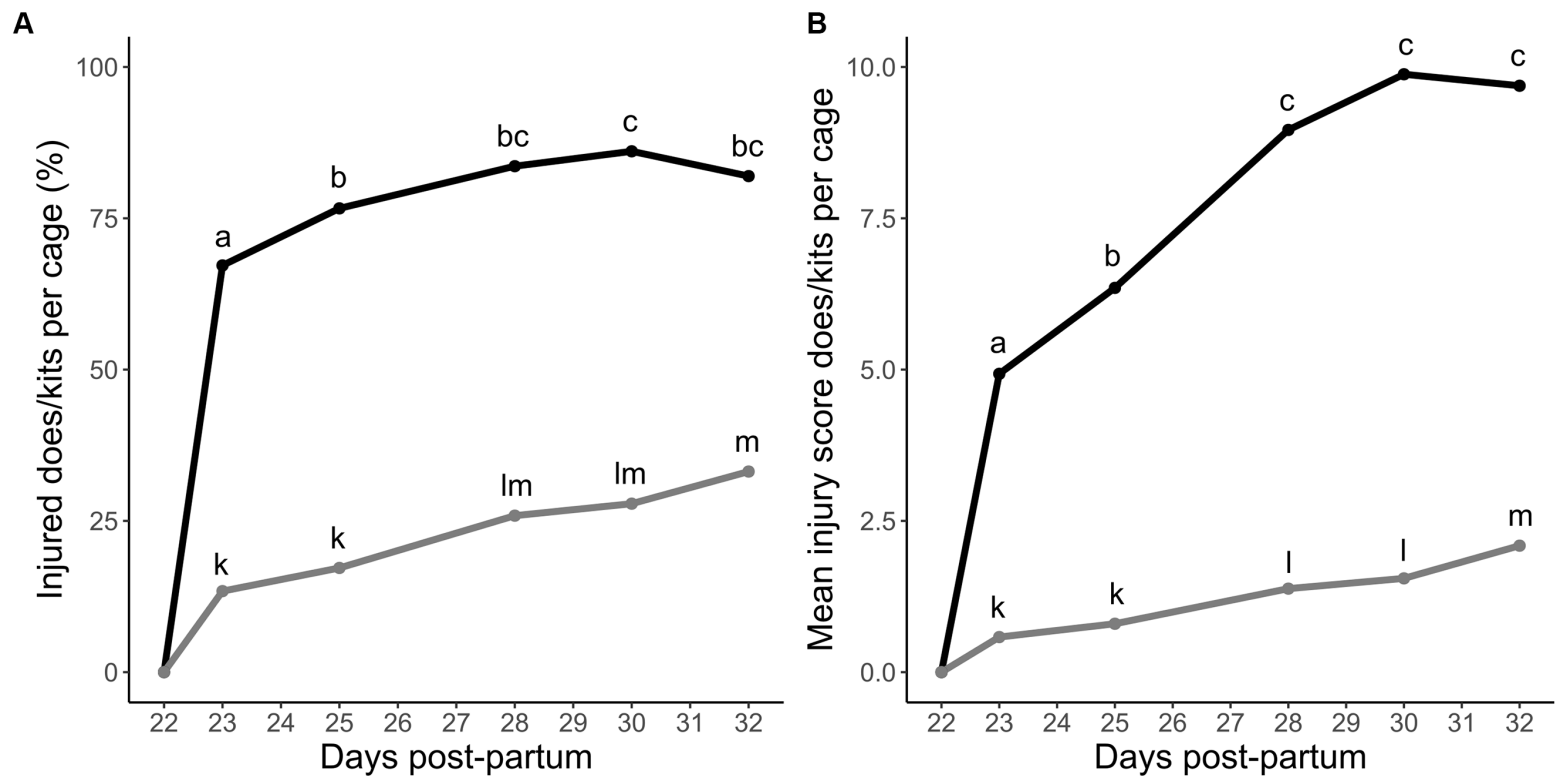
**(A)** Injured does and kits per multi-litter cage (%) on days 1, 3, 6, 8, and 10 after grouping. **(B)** Accumulation of doe and kit skin injury scores relative to the onset of grouping. The picture represents the average of summed skin injury scores per multi-litter cage (*N* = 60 cages) at 1, 3, 6, 8, and 10 days after grouping. Black and grey lines represent does and kits, respectively. Significant differences between days are represented by the superscripts ^a,b,c^ and ^k,l,m^ for does and kits, respectively, (pairwise *post-hoc* Tukey’s test). Injury scores were log(x + 1) transformed for analysis.

**Figure 4:**
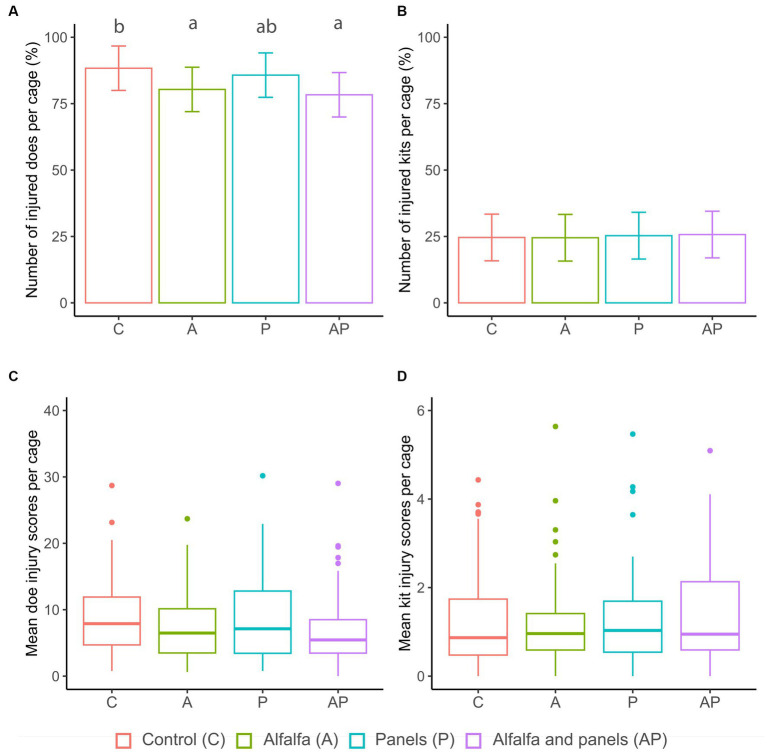
**(A,B)** Injured does and kits after grouping (% SD error bars). **(C,D)** Mean of summed doe and kit skin injury scores after grouping. The picture represents the values averaged per multi-litter cage (*N* = 15 per treatment) and observation day (1, 3, 6, 8, and 10 days after grouping). Significant differences between treatments are represented by superscripts ^a,b^ (pairwise *post-hoc* Tukey’s test).

The skin injuries acquired up to 10 days after grouping were most frequent on the trunk (65%), followed by the ears (17%) and head (7%). After grouping, half of the observed individual skin injuries ranged between a severity score of 2.5 and 2.7 on the t-VAS (median score = 2.6). The percentage of injured does after grouping was affected by observation day (*F_4,285_* = 12.18, *p* < 0.001) and treatment (*F_3,286_* = 4.76, *p* = 0.003). During the first day in groups, 67.2% of all does had acquired at least one new skin injury (Figure 3A). Relative to the first day, the percentage of injured does further increased significantly towards the third (76.6%, *p* = 0.01) and eighth day in the group (86.1%, *p* < 0.001, Figure 3A). On average, the percentage of injured does was higher in the control group (88.3%) compared to the A (80.4%, *p* = 0.04) and AP group (78.3%, *p* = 0.005, Figure 4A). On average, after grouping, 5.9% of the does had at least one newly acquired injury with a severity score equal to or higher than 5.0 on the t-VAS.

The doe injury severity score was significantly affected by day (*F_4,286_* = 39.73, *p* < 0.001, Figure 3B), but not by treatment (*F_3,287_* = 1.10, *p* = 0.36, Figure 4C). After an initial steep increase after the first day in the group, skin injury scores further increased significantly towards the third (*p* = 0.01) and sixth day in the group (*p* < 0.001) compared to the first day. After the sixth day, it stabilized (Figure 3B).

### 3.2 Skin injuries of kits

The skin injuries acquired by the kit after grouping were most frequent on the ears (55%), trunk (15%), and head (11%). After grouping, half of the observed individual skin injuries ranged between a score of 2.5 and 3.2 on the t-VAS (median score = 2.8). The percentage of kits that acquired an injury after grouping was significantly affected by observation day (*F_4,285_* = 29.29, *p* < 0.001). After the first and third day in the group, 13.4 and 17.2% of all kits acquired at least one skin injury that they did not have before the grouping (Figure 3A). Compared to the first day, this prevalence increased significantly towards the sixth (25.9%, *p* = 0.001) and tenth day in the group (33.2%, *p* = 0.004, Figure 3B). No significant treatment differences were found for the number of injured kits (*F_3,286_* = 0.15, *p* = 0.93, Figure 4B). On average, after grouping, 4.4% of the kits had at least one newly acquired injury with a score equal to or higher than 5.0 on the t-VAS.

The severity score of the kit injury was also significantly affected by observation day (*F_4,286_* = 60.32, *p* < 0.001) but not by treatment (*F_3,287_* = 0.10, *p* = 0.96, Figure 4D). The kit injury scores increased from the onset of group housing until the last day in the group (Figure 3B).

### 3.3 Behavior and activity scores

Most doe-doe agonistic behavior during the first 24h after grouping concerned fleeing/chasing behavior instead of aggressive displays (62.0 vs 38.0%). The fleeing and chasing behavior comprised 2.2% of the total observation time.

**Figure 5:**
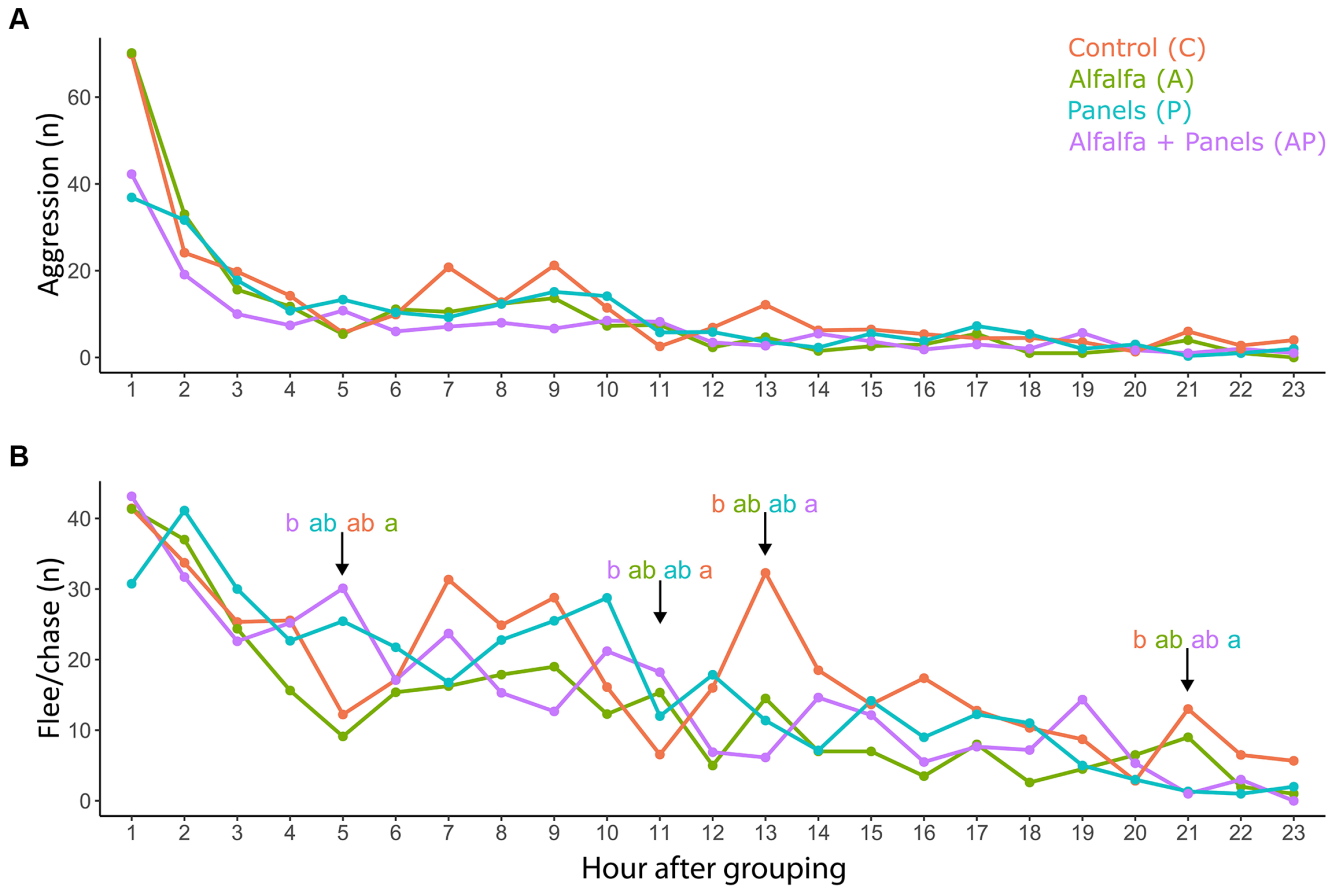
Doe agonistic behavior during the first 24 h after grouping: frequency (n) of aggressive **(A)** and flee/chase behavior **(B)**. The picture represents the values averaged per mutiple-litter cage (*N* = 9 per treatment). The dark period started on hour 7 and lasted until hour 19. Significant differences between treatments per hour are represented by superscripts ^a,b^ (pairwise post-hoc Tukey test). Analysis performed on log(x + 1) transformed data.

The averaged frequency of doe-doe aggression per multi-litter cage was affected by time (an hour after grouping, *F_21,582_* = 14.65, *p* < 0.001), but not by treatment (*F_2,601_* = 1.24, *p* = 0.31). The aggression declined rapidly during the first 2 h after grouping (Figure 5A). For the averaged frequency of flee/chase behavior, an interaction effect between treatment and time (hour) was observed (*F_65,539_* = 1.66, *p* = 0.002). It was higher for the AP treatment compared to the A treatment on hour 5 after grouping, and compared to the control group (*p* = 0.04) on hour 11. On hours 13 and 21, however, fleeing and chasing were more frequent in the control group compared to the AP (*p* = 0.001) and P treatment (*p* = 0.02) respectively (Figure 5B).

**Figure 6:**
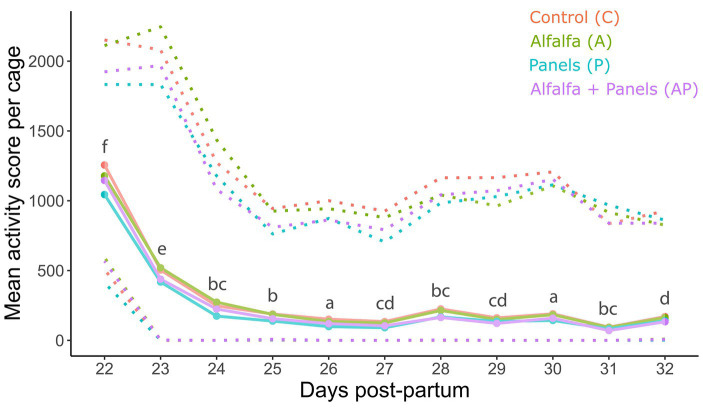
Rabbit activity scores (as a proxy for social unrest) averaged per multi-litter cage (*N* = 15 per treatment) treatment and observation day (1, 3, 6, 8, and 10 days after grouping). Higher activity scores represent a higher amount of movement and thus a higher rate of social unrest in the cage. Full lines in the picture represent mean activity scores, and upper and lower dashed lines represent maximum and minimum scores, respectively. Significant differences between days are represented by superscripts ^a,b,c,d,e,f^ (pairwise *post-hoc* Tukey’s test). Analysis performed on log(x + 1) transformed data.

No significant treatment difference was observed for the averaged scores of rabbit activity per multi-litter cage (*F_3,656_* = 0.53, *p* = 0.66, Figure 6). A significant time (day) effect, however, was present (*F_9,650_* = 213.72, *p* < 0.001). Rabbit activity declined rapidly during the first 2 days after grouping (Figure 6).

### 3.4 Correlations between skin injuries, behavior, and activity scores

The frequency of doe-doe aggression during the first day after grouping was significantly and positively associated with the skin injury score of the does, one day after grouping (*r* = 0.42, *p* = 0.01), but not with the injury scores of the kits (*r* = 0.02, *p* = 0.91). Similarly, the frequency of doe-doe flee/chase behavior was positively correlated with the injury scores of the does one day after grouping (*r* = 0.40, *p* = 0.01), but not with the kit injury scores (*r* = −0.07, *p* = 0.68).

No significant correlations were found between the mean or maximum activity score per cage and the injury scores of does or kits on any of the post-grouping days (Supplementary Table S1).

## 4 Discussion

In this study, the effects of the two types of cage enrichment (wooden panels and alfalfa) on skin injuries of the doe and kit, doe-doe agonistic behavior, and level of group activity (as a proxy for social unrest) were evaluated. The wooden panels were hypothesized to directly reduce the frequency of aggressive interactions by offering hiding places. The alfalfa blocks were introduced as foraging enrichment to distract does from fighting at the onset of group housing.

Based on the previous part-time group housing studies on breeding does, a steep increase in the number of injured does on the first day after grouping was expected (21, 24–26) and it was also confirmed in the present study (67.2% of the does get injured shortly after grouping). The number of injured does increased significantly towards the third and eighth day after grouping relative to the first day, but these increases were less steep. Similarly, after grouping five does with their kits in multi-litter cages, Rommers and de Greef (27) reported no decrease in the average skin injury score of the doe between days 4 and 13. This was contrary to their expectations, as an earlier pilot study had found less severe injuries at the end of the group-housing phase compared to a few days after grouping (28). Furthermore, in the study of Huang et al. (29), the severity of skin injuries increased as group housing progressed. However, the scoring method for the severity of skin injury used in the present study did not differentiate between old and fresh wounds and could therefore not detect a possible decrease in the number of new injuries. Furthermore, the absence of a significant increase in the injury scores of the does toward the end of the group housing suggests that agonistic behavior has decreased. The decline in rabbit activity also supports this. Based on the action clips, it seemed that the agonistic interactions were highest shortly after grouping but declined fast during the subsequent days. Furthermore, aggression and flee/chase behavior were also highest in the first hours after grouping but declined afterward. Based on other studies, it can be assumed that the agonistic behavior would further decrease towards the end of the group-housing phase (30). When studying the agonistic behavior of the does in groups of four part-time group-housed does between days 18 and 39 pp., Buijs et al. (31) found that a larger proportion of the observed time was spent on evasive (fleeing and retreating) and offensive (attacking and chasing) behaviors on the first day after grouping compared to the fourth and twelfth day. In the present study, however, the increase in the mean injury scores and the percentage of injured kits indicated that kit-directed aggression may have persisted. It is unlikely that kits exhibited aggression toward other kits [as opposed to sexually mature rabbits, (32)], suggesting that kit lesions were caused by the does.

The percentage of injured does was significantly lower in the A and AP treatments compared to the control treatment. The alfalfa, provided throughout the entire group housing phase, may have distracted does from fighting which would explain the lower prevalence of injured does. This treatment effect, however, was absent for the mean injury score of the does. Furthermore, for the kits, no treatment effects on either the percentage of injured kits or the injury score of the kits were found. In the pilot study of Rommers et al. (33), wooden panels and plastic pipes were offered to provide fleeing and hiding options for does. The authors concluded that the panels offered the best opportunity for escape if aggression between does occured. In the present study, however, wooden panels had no effect on injuries unless combined with alfalfa. The lack of a significant effect of panels (P treatment) suggests that the lower percentage of injured does in the AP treatment may be attributed due to the presence of alfalfa rather than the wooden panels. The alfalfa blocks seemed to be attractive for the rabbits as they were consumed and replenished throughout the group housing phase. Previous studies have also shown positive effects of consumable cage enrichment, such as the introduction of gnawing blocks, which reduced nest box inspections by does and decreased nervousness in fattening rabbits (34, 35). In the study of Birolo et al. (36), growing rabbits housed in groups exhibited active and positive responses toward new environments and objects when their cages were provided with compressed hay blocks.

However, regardless of the enrichment treatments, after grouping, the injuries with a score above 5.0 on the t-VAS (corresponding with deeper lesions and longer scratches) were found on does (5.9%) and kits (4.4%). Although some levels of aggression and hence skin injuries are hard to avoid when creating new groups of breeding does, severe skin injuries are a serious animal welfare concern and are unacceptable. If significant improvements cannot be achieved, to minimize severe aggression in group housing systems, the suitability of housing does in social groups as an alternative to single-litter housing may be questioned. Nevertheless, it is crucial to explore additional approaches to house the does in groups, considering the importance of social interactions. An extension of the reproductive cycle increases the number of days that does could be housed in groups. A longer period of group housing may provide more opportunities for social interactions after a hierarchy is established. However, this would implicate fewer kits per doe per year, and thus reduce the income for the farmer. Furthermore, between reproduction cycles, the group composition rarely remains stable in a commercial management procedures. This implies that many does are unacquainted at the start of a new group-housing period such that the dominance relations need to be formed from scratch. As suggested by Rommers and de Greef (27), ‘preparing’ does by housing them in groups with other does before their first litter could be beneficial. However, in part-time group housing systems, it needs to be confirmed if does will remember and respect the hierarchy from the previous reproduction cycle. More information on the effect of familiarity and acquaintance is needed before practical recommendations can be made. The research should continue to develop alternative housing or management systems that accommodate the social and behavioral needs of breeding does without severely compromising their physical health or integrity.

## 5 Conclusion

When housing groups of four does with their litters in multi-litter cages between day 22 and 32 pp., no profound effects of cage enrichment (wooden panels and alfalfa) were found on the skin injuries of the does and kits, doe aggressive behavior, and rabbit activity (as a proxy for agonistic behavior). The percentage of injured does were slightly lower in the A and AP treatments, as compared to the control treatment, indicating that alfalfa may somewhat distract does from fighting. The agonistic behavior of the does decreased a few hours after grouping in all treatments. Overall, the activity (as a proxy for social unrest) declined most significantly during the first 3 days in the group. The high prevalence of (severely) injured animals, even in the enriched multi-litter cages, indicates that more effective or additional strategies are needed to reduce welfare problems associated with aggression and unrest when does are grouped, and to establish a social hierarchy.

## Data availability statement

The datasets presented in this study can be found in online repositories. The names of the repository/repositories and accession number(s) can be found at: https://www.researchportal.be.

## Ethics statement

The animal study was approved by Ethics Committee for the Use of Animals in Research of Flanders Research Institute for Agriculture, Fisheries and Food (ILVO). The study was conducted in accordance with the local legislation and institutional requirements.

## Author contributions

LD: Writing – review & editing, Writing – original draft, Visualization, Resources, Project administration, Methodology, Investigation, Formal analysis, Data curation, Conceptualization. NI: Writing – review & editing, Visualization, Software, Formal analysis, Data curation. JV: Writing – review & editing, Supervision. ED: Writing – review & editing, Supervision, Methodology, Funding acquisition. FT: Writing – review & editing, Supervision, Methodology, Funding acquisition.
