# Supplementary material for: Cage enrichment to minimize aggression in part-time group-housed female breeding rabbits
Source: Front Vet Sci. 2024 Jun 4;11:1401021. doi: 10.3389/fvets.2024.1401021 (PMC11185069; doi:10.3389/fvets.2024.1401021)
Supplement: Supplementary file 1 [file Table_1.docx]

**Supplementary table 1.** Spearman’s rank correlation (*r*) between mean and maximum activity values and doe and kit injury scores on day (d) 1, 3, 6, 8 and 10 after grouping (*N* = 15 multi-litter cages per treatment).

|  | Mean activity | |  | Maximum activity | |
| --- | --- | --- | --- | --- | --- |
|  | *r* | *P-value* |  | *r* | *P-value* |
| Doe injury score d1 | -0.12 | 0.35 |  | -0.04 | 0.75 |
| Kit injury score d1 | -0.06 | 0.66 |  | -0.06 | 0.67 |
| Doe injury score d3 | 0.14 | 0.29 |  | 0.08 | 0.55 |
| Kit injury score d3 | -0.03 | 0.83 |  | -0.11 | 0.43 |
| Doe injury score d6 | -0.03 | 0.85 |  | 0.07 | 0.59 |
| Kit injury score d6 | -0.18 | 0.17 |  | -0.22 | 0.09 |
| Doe injury score d8 | 0.04 | 0.76 |  | -0.02 | 0.87 |
| Kit injury score d8 | -0.06 | 0.63 |  | -0.03 | 0.84 |
| Doe injury score d10 | 0.03 | 0.85 |  | 0.08 | 0.54 |
| Kit injury score d10 | -0.06 | 0.63 |  | -0.09 | 0.50 |
